# Comparison of Odontogenic Differentiation of Human Dental Follicle Cells and Human Dental Papilla Cells

**DOI:** 10.1371/journal.pone.0062332

**Published:** 2013-04-19

**Authors:** Lijuan Guo, Jie Li, Xiangchen Qiao, Mei Yu, Wei Tang, Hang Wang, Weihua Guo, Weidong Tian

**Affiliations:** 1 State Key Laboratory of Oral Diseases, West China Hospital of Stomatology, Sichuan University, Chengdu, P.R. China; 2 Department of Oral and Maxillofacial Surgery, West China School of Stomatology, Sichuan University, Chengdu, P.R. China; 3 College of Life Science, Sichuan University, Chengdu, P.R. China; 4 Department of Pedodontics, West China School of Stomatology, Sichuan University, Chengdu, P.R. China; Indian Institute of Toxicology Research, India

## Abstract

Classical tooth development theory suggests that dental papilla cells (DPCs) are the precursor cells of odontoblasts, which are responsible for dentin development. However, our previous studies have indicated that dental follicle cells (DFCs) can differentiate into odontoblasts. To further our understanding of tooth development, and the differences in dentinogenesis between DFCs and DPCs, the odontogenic differentiation of DFCs and DPCs was characterized *in vitro* and *in vivo*. DFCs and DPCs were individually combined with treated dentin matrix (TDM) before they were subcutaneously implanted into the dorsum of mice for 8 weeks. Results showed that 12 proteins were significantly differential, and phosphoserine aminotransferase 1 (PSAT1), Isoform 2 of hypoxia-inducible factor 1-alpha (HIF1A) and Isoform 1 of annexin A2 (ANXA2), were the most significantly differential proteins. These proteins are related to regulation of bone balance, angiogenesis and cell survival in an anoxic environment. Both DFCs and DPCs express odontogenic, neurogenic and peridontogenic markers. Histological examination of the harvested grafts showed that both DFCs and DPCs form pulp-dentin/cementum-periodentium-like tissues *in vivo*. Hence, DFCs and DPCs have similar odontogenic differentiation potential in the presence of TDM. However, differences in glucose and amino acid metabolism signal transduction and protein synthesis were observed for the two cell types. This study expands our understanding on tooth development, and provides direct evidence for the use of alternative cell sources in tooth regeneration.

## Introduction

Tooth organogenesis involves a series of interactions between the oral epithelium and the underlying cranial neural crest-derived cells [1]-[3]. Once the thickening of the oral epithelium occurs, oral epithelium cells begin to differentiate into odontogenic epithelial cells, while mesenchymal cells begin to differentiate into odontogenic mesenchymal cells, e.g. dental papilla cells (DPCs) and dental follicle cells (DFCs). Previous studies suggested that DFCs and DPCs possess osteogenic, adipogenic and neural differentiation potential, which indicates that these two cell lineage maintain stem cell-like properties [4].

The formation of primary dentine initiates the development of cementum and enamel. The “sandwich” structure of dental follicle, dentin, and dental papilla tissues enables DFCs and DPCs to contact the dentin matrix. During the late bell stage of tooth development, DPCs differentiate into preodontoblasts as result of induction by mature ameloblasts, and preodontoblasts produce the predentin. Once DPCs contact predentine, they begin to differentiate into odontoblasts inside the predentin, and odontoblasts produce the primary dentin. Odontoblasts secrete the extracellular matrix and gradually move backward. The secreted extracellular matrix gradually mineralizes and becomes primary dentin. Meanwhile, the formation of Hertwig’s epithelial root sheath (HERS) initiates the development of the tooth root. The fracture of HERS enables DFCs to contact the dentin and leads DFCs to differentiate along periodontal lineages. Hence, the differentiation of both DFCs and DPCs is influenced by dentin matrix. According to the classic tooth development theory, DPCs are the precursors of odontoblasts and they are also responsible for predentin secretion and dentin maturation. Interestingly, our previous studies [5]-[7] indicated that DFCs could differentiate into odontoblasts and subsequently form dentin tissue. However, the differences in dentinogensis of DFCs and DPCs are still unclear. Additionally, our previous studies indicate that DFCs are perhaps suitable seed cells for regeneration of dentin tissues and tooth root. This is based on the finding that DFCs (P = 30) still maintain strong potential for tissue regeneration [5], [8]. Most importantly, DFCs can be easily obtained from impacted wisdom tooth and propagated under laboratory conditions. However, unlike DFCs, DPCs are difficult to harvest because the tooth papilla is only present in the tooth germ during the crown-forming stage. A comparative study of dentinogenic characteristics of DFCs and DPCs may provide evidence for replacing DPCs by DFCs as seed cells for dentin and dental pulp regeneration.

Our previous studies found that treated dentin matrix (TDM) could maintain functional proteins and factors in nature dentin matrix, which make TDM qualified to simulate the induced micro-environment as the natural dentin. Therefore, to explore the differences in odontogenic differentiation ability of DFCs and DPCs, we harvested DFCs and DPCs from human impacted teeth at the crown-forming stage, and performed a comparative investigation of their biological characteristics under the effects of TDM *in vitro* and *in vivo*. In addition, this study is the first to compare the proteome of DFCs and DPCs. These findings expand our understanding of the role of DFCs and DPCs in tooth development, and provide evidence for using odontogenic mesenchymal cells as seed cells for tooth tissue regeneration.

## Materials and Methods

### Isolation and culture of DFCs and DPCs

According to the patients' panoramic radiographs, tooth germs of the impacted third molar at the crown-forming stage were collected from patients (n = 10, 16-17 years of age) with written consent signed by parents during orthodontic treatment in the West China Stomatology Hospital. All experiments were conducted in accordance with the ethical protocol approved by the Committee of Ethics of the Sichuan University. DFCs and DPCs were derived as previously described [8], [9]. Briefly, DFCs and DPCs were isolated from dental follicle and dental papilla, and cultured in α minimum essential medium (α-MEM, Gibco BRL, Gaithersburg, MD, USA), supplemented with 10% fetal bovine serum (FBS, Gibco BRL), 0.292 mg/ml glutamine (Sigma, St. Louis, MO, USA), 100 U/ml penicillin (Sigma) and 100 µg/ml streptomycin (Sigma) in a humidified atmosphere at 37°C with 5% CO_2_. The medium was changed every three days [7], [8], [10].

### Morphological characterization of cells

The cells were characterized using optical microscopy (Nikon, Tokyo, Japan), transmission electron microscopy (TEM) (JEM 100 SX, Jeol, Tokyo, Japan) and fluorescence microscopy (Leica Optical, Wetzlar, Germany) as described previously [7], [8].

*Light microscopy*

DFCs and DPCs were seeded separately in culture plates (BD, Franklin Lakes, NJ, USA) separately at a cell density of 5×10^4^ cells/ml, and supplemented with α-MEM with 10% FBS. Images were captured using phase-contrast inverted microscope (Nikon) when the cells had reached 80% confluency. At least three samples were prepared for each experiment.

#### Ultrastructural characterization

DFCs and DPCs were pelleted separately at 3000×g for 5 min at 4°C and fixed in 3% glutaraldehyde (Sigma) in 0.1 M cacodylate buffer (pH 7.3) (Sigma) for 1 h. After dehydrating them by subjecting them to a graded ethanol series (50, 70, 95 and 100%), the cells were resin embedded using Epon 812 (Sigma). The resulting ultra-thin sections were stained with uranyl acetate and lead citrate (Sigma). The sections were observed using transmission electron microscopy (JEM 100 SX, Jeol). The experiment was repeated at least three times.

#### Expression of marker-proteins

DFCs and DPCs were seeded onto coverslips (18×18 mm^2^) at a cell density of 5×10^4^ cells/ml and cultured for 24 h. Cells were fixed in 4% polyoxymethylene for 10 minutes. The subsequent steps for cells staining were performed according to the manufacturer’s instructions. The primary antibodies against the following proteins were used: STRO-1 (1∶100; R&D, Minneapolis, MN, USA), a marker for stromal stem cells [11]; Vimentin (1∶100; Thermo Scientific, Waltham, MA, USA), a marker for mesenchymal cells[12], and cytokeratin-14 (CK-14;1∶150; Abcam, Cambridge, MA, USA), a marker for epithelial cell. Phosphate buffered saline (PBS) was used instead of the primary antibody as a negative control. Secondary antibodies were conjugated to FITC/TRITC and used at a concentration of 1∶100 (Santa Cruz Biotechnologies, Inc., CA, USA). DAPI (Sigma) was used at a concentration of 1∶1000 for counterstaining. Samples were examined using a fluorescence microscope (Leica Optical).

### Biological characteristics of DFCs and DPCs

To explore the biological characteristics of DFCs and DPCs, ultrastructural studies, Bromodeoxyuridine (BrdU) cell proliferation assay, cell growth curve analysis, telomerase activity, cell surface antigen analysis, and multipotential differentiation capacity experiments were performed.

#### Ultrastructural comparison

DFCs and DPCs were processed as described in *Ultrastructural characterization*.

#### Cell proliferation

5×10^5^ DFCs or DPCs were seeded onto coverslips (18×18 mm^2^) and cultured for one day. To keep the majority of cells in the G_0_ phase, α-MEM (supplemented with 0.4% FBS) was added to cells to synchronize cell growth for three days. Subsequently, BrdU was added to the medium at a final concentration of 10 µM, cultured for 40 min at 37°C. The cells were then washed three times with PBS, and fixed in 4% paraformaldehyde for 30 min. Resulting cells were treated with 2 mol/L HCl at 37°C for 60 min and then neutralized with 0.1 mol/L sodium borate (pH 8.5) for 10 min. The cells were then treated as described previously in the section on ‘expression of marker-proteins’. Anti-Bromodeoxyuridine antibody was used for cell labeling (Millipore, Billerica, MA, USA).

#### Cell growth curve

Cell viability was determined using Cell Counting Kit-8 (CCK-8, Tokyo, Japan). DFCs and DPCs were cultured in 96-well plates (Becton Dickinson) at a cell density of 5×10^4^ cells/ml. 100 µl α-MEM medium (supplemented with 10% FBS) and 10 µl CCK-8 solution were added to each well at each time-point (day 1, 2, 3, 4, and 5). After 3 hours of incubation with the kit reagent at 37°C, the absorbance of 100 µl solution of each sample (n = 6) was examined at 450 nm using a spectrophotometer (Thermo Scientific Varioskan Flash, Thermo Scientific).

#### Telomerase activity

Cell monolayers were washed three times with PBS before they were lysed using cell lysis buffer, containing 0.1% protease inhibitor, 0.5% phosphatase inhibitor and 0.5% 100 mM phenylmethanesulfonyl fluoride (PMSF). Proteins were quantified using modified Bradford Protein Assay (Bio-Rad, Hercules, CA, USA). 50 µg of total protein from cell lysates was separated by sodium dodecyl sulfate polyacrylamide gel electrophoresis (SDS-PAGE), transferred to nitrocellulose membranes (Invitrogen, Carlsbad, CA, USA) and probed with anti-TEP1 antibody (Rockland, Gilbertsville, PA, USA) at a concentration of 1∶400. After conjugation with secondary peroxidase antibody (Jackson ImmunoResearch Laboratories, USA), the membranes were visualized with a chemiluminescent horseradish peroxidase (HRP) substrate (Millipore) using ChemiDoc XRS Systems (Bio-Rad). All experiments were performed in triplicate.

#### Cell surface antigen analysis

5×10^6^ DFCs or DPCs were incubated with a FITC/PE/APC-conjugated primary antibody for 30 min at room temperature prior to being analyzed on a FACS Calibur flow cytometer (FACS) (BD). The antibodies used included: APC mouse anti-human CD29 (1∶100; BioLegend, San Diego, CA USA), FITC mouse anti-human CD31 (1∶120; BioLegend), FITC mouse anti-human CD44 (1∶100; BD), FITC mouse anti-human CD90 (1∶150; BD), PE mouse anti-human CD106 (1∶100; BD), PE mouse anti-human CD146 (1∶100; BD), and FITC mouse anti-human STRO-1 (1∶100; R&D). Mouse IgG (1∶100; Beckman Coulter, Brea, CA, USA) was used as an isotype control.

#### Detection of multipotential differentiation capacity

DFCs and DPCs (5×10^4^ cells/ml) were loaded in 6-well plates (BD) and cultured in α-MEM supplemented with 10% FBS. After the cells had reached 80% confluency, osteogenic medium (α-MEM supplemented with 10% FBS, 5 mM <beta>-glycerophosphate, 100 nM dexamethasone, and 50 µg/ml ascorbic acid [7]) or adipogenic medium (α-MEM supplemented with 10% FBS, 2 µM insulin, 0.5 mM isobutyl-methylxanthine, and 10 nM dexamethasone [13]) were used for the following 25 days. α-MEM supplemented with 10% FBS was used in the control group.

The cell culture medium was changed every three days. After 25 days, the cells were washed twice with PBS and fixed in 4% paraformaldehyde for 30 min. Osteogenic cultures were stained with alizarin red (Sigma). Adipogenic cultures were stained with Oil red O (Sigma). The cells were imaged using phase-contrast inverted microscope (Nikon). The nodule formation and lipid areas were quantitatively measured using image analysis system (Image-Pro Plus 5.0; Media Cybernetics, Inc., Rockville, MD, USA).

### Differentially expressed proteins in DFCs and DPCs

#### Two-dimensional Electrophoresis

3×10^7^ DFCs and DPCs were respectively lysed in 1 ml lysis buffer containing 7 M urea (Bio-Rad), 2 M thiourea (Sigma), 4% 3-[(3-cholamidopropyl) dimethylammonio]-1- propanesulfonate (CHAPS; Bio-Rad), 100 mM dithiothreitol (DTT) (Bio-Rad), 0.2% ampholyte (pH 3-10, Bio-Rad), and protease inhibitor cocktail 8340 (Sigma). The cells were chilled on ice and sonicated (5 s sonication with 10 s interval) six times at 30% amplitude. After centrifugation (14000×rpm, 1h, 4°C), supernatants were collected and protein concentration was determined using the RC DC Protein Assay Kit (Bio-Rad). Protein samples (2 mg) were applied to immobilized pH gradient (IPG) strip (17 cm, pH 3-10, Bio-Rad) using a passive rehydration method. After 12-16 h of rehydration, the strips were transferred to a PROTEAN*®* IEF Cell (Bio-Rad). Isoelectric Focusing (IEF) was performed (250 V for 30 min, linear; 1000 V for 1 h, rapid; linear ramping to 10000 V for 5 h, and finally 10000 V for 6 h). The strips were then equilibrated in equilibration buffer (25 mM Tris-HCl, pH 8.8, 6 M urea, 20% glycerol, 2% SDS, and 130 mM DTT) for 15 min, followed by another 15 min in the equilibrium buffer in which DTT was replaced with 200 mM iodoacetamide. Electrophoresis in the second dimension was performed using 12% SDS-PAGE at 30 mA constant current per gel. The resulting gels were stained with Coomassie Brilliant Blue (CBB) R-250 (Merck, Germany) and scanned using Bio-Rad GS-800 scanner. The protein maps were analysed by PD-Quest software Version 8.0 (Bio-Rad). The protein spots on each gel were normalized as the percentage of total spots and evaluated in terms of optical density. Only proteins spots that changed consistently and significantly (>1.5-fold) were selected for Mass Spectrometry (MS) analysis.

#### In-gel digestion

In-gel protein digestion was carried out using In-Gel Tryptic Digestion Kit (Thermo Scientific) according to the manufacturer’s instructions. Briefly, spots were cut out from the gel (1-2 mm diameter) using a razor blade, and destained twice with 200 µl Destaining Solution at 37°C for 30 min. Then, 30 µl of Reducing Buffer was added to cover the gel slices which were incubated at 60°C for 10 minutes. After the removal of the Reducing Buffer, 30 µl Alkylation Buffer was added to the tube, followed by 1 h incubation in the dark at room temperature. Subsequently, Alkylation Buffer was discarded; samples were rinsed twice in 200 µl Destaining Buffer (37°C, 15 minutes) with shaking. After reduction and alkylation, the gel slices were incubated in 50 µl acetonitrile for 15 minutes at room temperature. After drying, the gels were pre-incubated for 15 minutes in 10-20 µl Activated Trypsin solution at room temperature. Then, 25 µl Digestion Buffer was added to the gels, followed by overnight incubation at 30°C. Tryptic digests were extracted using 10 µl of 1% trifluoroacetic acid (TFA) for 5 minutes. The combined extracts were dried in a speed-VAC concentrator (Thermo Scientific) at 4°C. The samples were then subjected to mass spectrometry.

#### Matrix-assisted laser desorption-ionization time-of-flight mass spectrometry (MALDI-TOF-MS)

The tryptic peptides were mixed in R-cyano-4-hydroxycinnamic acid matrix solution. One microliter of the mixture was analyzed using Voyager System DE-STR 4800 Mass Spectrometer (Applied Biosystems, Carlsbad, CA, USA) to obtain a peptide mass fingerprint (PMF). For searching the PMF map database, Mascot Distiller was used to obtain the monoisotopic peak list from the raw mass spectrometry files. Peptide matching and protein searches against IPI.HUMAN.v3.52 database were performed using the GPS Explorer software (Applied Biosystems) with mass tolerance of 50 ppm. For tandem mass spectrometry database query, the peptide sequence tag (PKL) format file generated from MS/MS was imported into the Mascot search engine with MS/MS tolerance of ±0.3 Da to search the IPI HUMAN.v3.52 database. The proteins with scores>60 were considered to be positively identified(*p*<0.05).

### Odontogenic differentiation of DFCs and DPCs *in vitro*

TDM was used to provide the odontogenic microenvironment for DFCs and DPCs, the odontogenic differentiation abilities of DFCs and DPCs were determined accordingly *in vitro* [6], [13]. Scanning electron microscope (SEM), quantitative real time PCR (qRT-PCR) and western blotting were used to characterize the growth of cells.

#### Preparation of TDM

TDM was prepared according to a well-established protocol [6], [13]. Briefly, maxillary premolars were harvested from patients who required their removal for clinical reasons at the West China Stomatology Hospital of Sichuan University. Outer cementum and part of the dentin was removed by grinding according to the tooth profile. Dental pulp tissues and predentin were removed using mechanical means. The resulting human dentin matrix was soaked in deionized water for 5 hours and mechanically cleaned for 20 minutes every hour using an ultrasonic cleaner. The deionized water was changed every hour. Human dentin matrices were then respectively soaked first in 17%, then in 10% and finally in 5% EDTA (Sigma). The samples were then maintained in sterile PBS (Hyclone) with 100 units/ml penicillin (Hyclone) and 100 µg/ml streptomycin (Hyclone) for 72 hours, washed in sterile deionized water for 10 minutes in an ultrasonic cleaner, and were finally stored in α-MEM (Hyclone) at 4°C.

#### SEM

5×10^4^ DFCs and DPCs were respectively seeded onto TDMs. The resulting cell-TDM constructs were cultured in α-MEM supplemented with 10% FBS. At each time-point (day 1, 3, 5 and 7), cells were washed three times in PBS before being fixed in 2.5% glutaraldehyde at 4°C for 24 h. Dehydration of samples was performed in a CO_2_ critical-point dryer. Samples were observed by SEM. All samples were examined in triplicates.

#### RNA preparation and qRT-PCR analysis

DFCs and DPCs (5×10^4^) were seeded onto TDMs. The resulting cell-TDM constructs were cultured in α-MEM supplemented with 10% FBS for 7 days. The cells were divided into four groups: DFCs, DPCs, DFCs seeded onto TDMs (induced DFCs, iDFCs), DPCs seeded onto TDMs (induced DPCs, iDPCs). Total RNA was obtained using RNAiso™ Plus (TaKaRa Biotechnology, Tokyo, Japan). cDNAs were synthesized from the extracted RNA with PrimeScript*®* RT reagent Kit Perfect Real Time (TaKaRa Biotechnology). Relative expression of genes quantified via real-time PCR using SYBR*®* Premix Ex Taq™ (Perfect Real Time) (TaKaRa Biotechnology) using an ABI Prism 7300 System (Applied Biosystems). The PCR conditions were: 1 cycle, 95°C for 30 seconds; 40 cycles, 95°C for 5 seconds and 60°C for 31 seconds; the last cycle 95°C for 15 seconds, 60°C for 1 minute, and 95°C for 15 seconds. Dissociation curves were used to verify primer specificity. D-glyceraldehyde-3-phosphate- dehydrogenase (GAPDH) was used as an internal reference and relative mRNA levels were quantified using the 2^−ΔΔC^T method [14]. Primer sequences for GAPDH, dentin sialophosphoprotein (DSPP), dentin matrix protein 1 (DMP-1), tubulin, neurofilament (NF), type I collagen (COL-1), alkaline phosphatase (ALP), osteopontin (OPN), bone sialoprotein (BSP), periostin and transforming growth factor β1 (TGF-β1) are listed in Table 1. The experiment was performed three times.

**Table 1:** Oligonucleotide primer sequences utilized in the quantitative real-time PCR analysis.

Target cDNA	Primer sequence (5′-3′)	Product size (bp)	NBCI no.
GAPDH	F CTTTGGTATCGTGGAAGGACTCR GTAGAGGCAGGGATGATGTTCT	132	NM_002046.3
DSPP	F CTGTTGGGAAGAGCCAAGATAAGR CCAAGATCATTCCATGTTGTCCT	129	NM_014208.3
DMP-1	F GTGAGTGAGTCCAGGGGAGATAAR TTTTGAGTGGGAGAGTGTGTGC	111	NM_004407.3
Tubulin	F AGCAAGGTGCGTGAGGAGTATCR GCAGTAGGTCTCATCCGTGTTCT	147	BC000748.2
NF	F TACCAGGAAGCCATTCAGCAGR CCAAAGCCAATCCGACACTCT	170	AF203032.1
Col-1	F AACATGGAGACTGGTGAGACCTR CGCCATACTCGAACTGGAATC	145	NM_000088.3
ALP	F TAAGGACATCGCCTACCAGCTCR TCTTCCAGGTGTCAACGAGGT	170	NM_000478.4
OPN	F CAGTTGTCCCCACAGTAGACACR GTGATGTCCTCGTCTGTAGCATC	127	J04765.1
BSP	F GATTTCCAGTTCAGGGCAGTAGR CCCAGTGTTGTAGCAGAAAGTG	169	NM_004967.3
TGF-β1	F GTGGACATCAACGGGTTCACTACR GTGGAGCTGAAGCAATAGTTGG	167	BC022242.1
Periostin	F TGGAGAAAGGGAGTAAGCAAGGR TTCAAGTAGGCTGAGGAAGGTG	134	NM_001135934.1

#### Western blotting analysis

DFCs and DPCs (5×10^4^) were seeded onto TDMs, and the resulting cell-TDM constructs were cultured in α-MEM supplemented with 10% FBS for 7 days. The protocol for the preparation of protein samples and western blotting was described previously in the section on ‘Telomerase activity’. The antibodies used for western blotting were anti-β-actin (1∶500; Abcam), anti-dentin sialoprotein (DSP, 1∶200; Santa Cruz biotechnology), anti-DMP1 (1∶200; Santa Cruz biotechnology), anti-NF (1∶1000; Millipore), anti-β-Tubulin III (1∶1000; Millipore), anti-ALP (1∶500; Abcam), anti-BSP (1∶100; Abcam), anti-OPN (1∶1000; Abcam), anti-COL1 (1∶500; Abcam), anti-periostin (1∶500; Abcam) and anti-TGFβ1 (1∶400; Abcam).

### *In vivo* studies on the odontogenic differentiation of DPCs and DFCs

DFCs and DPCs (5×10^4^) were seeded on TDM and cultured *in vitro* for three days during which the cells adhered and proliferated on the TDM and covered the dentinal tubules before they were implanted into the dorsum of nude mice (*BALB/c nude mice*). All animal experiments were conducted in accordance with the ethical protocol approved by the Committee of Ethics of the Sichuan University for animal experiments. Nude mice were obtained from the Laboratory Animal Research Centre of Sichuan University, and were housed on a daily ration of Purina rodent chow in housing quarters with cycled light (12 h on/off), regulated temperature, and sterile water. Six nude mice were divided into two groups: DFCs group (TDM combined with DFCs) and DPCs group (TDM combined with DPCs). Cell/TDM constructs were subcutaneously implanted into the dorsum of mice for 8 weeks. A natural mandibular third molar was used as control. Implants were fixed in 4% paraformaldehyde at 4°C for 48 h, and demineralized in 10% EDTA (pH 6.9) for two months before they were paraffin embedded and sectioned (4 µm). Sections were histologically examined using hematoxylin and eosin staining (H&E) and Masson staining. Immunohistochemistry was used to detect if the markers related to dental pulp and periodontium were present in the newly formed tissues. Primary antibodies used for immunohistochemistry: DSP (Santa Cruz biotechnology), anti-Factor VIII (Santa Cruz biotechnology), anti-periostin (Abcam) and anti-human mitochondria (Millipore) were used at a dilution of 1∶100 according to the manufacturer’s instructions. DSP was used to identify newly-formed dentin tissue, anti-Factor VIII was used for the identification of neovascularization, periostin was used to identify neocementum-periodentium-like tissue, and anti-human mitochondria antibodies were used to identify human derived seed cells in the newly-formed tissues. The experiments were performed at least three times. The images were captured using microscopy (Leica Optical).

### Statistical analysis

Data were gathered at least in triplicate, and expressed as mean ± standard deviation (SD). A paired *t*-test analysis of variance was used to analyze differences between groups. *p*<0.05 was considered significant for all analyses. Computations were performed using the SPSS version 11.5 software.

## Results

### Isolation and identification of DFCs and DPCs

**Figure 1:**
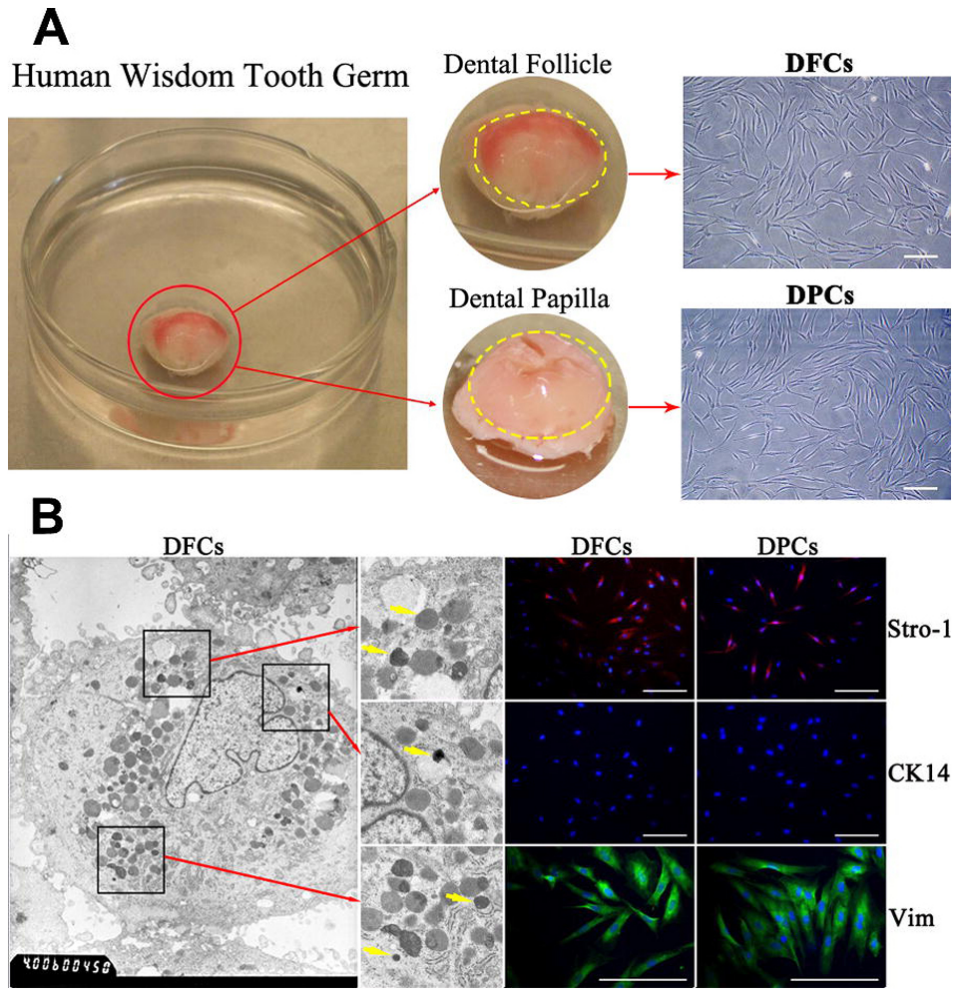
Culture and identification of DFCs and DPCs. (A) Primary DFCs and DPCs from human wisdom tooth germ. The dental follicle on the crown surface has visible blood supply. The dental papilla located inside the crown is transparent and jelly-like. Both DFCs and DPCs show typical fibroblast-like spindle morphology under a light microscope. (B) Homogeneous electron-dense granules in DFCs indicated by yellow arrows are featured for DFCs. Both cells were positive for STRO-1 and for Vimentin, but negative for epithelial marker CK-14. Scale bar  =  100 µm in (A) and (B).

Dental follicle and dental papilla were harvested from human wisdom tooth germ (Fig. 1A). DFCs and DPCs were extracted and expanded from human dental follicle and dental papilla tissues (Fig. 1A). Both cells exhibited spindle-shape fibroblast-like morphology (Fig. 1A). TEM demonstrated that DFCs were rich in rough endoplasmic reticulum (RER) and lysosomes (Fig. 1B). Electron-dense granules were observed in the vicinity of RER and lysosomes of DFCs (Fig. 1B). Both cell phenotypes were negative for CK-14, an epithelial cell marker, and positive for Vimentin, a mesenchymal cell marker, and STRO-1, a mesenchymal stem cells marker (Fig. 1B).

### Ultrastructural comparison

**Figure 2:**
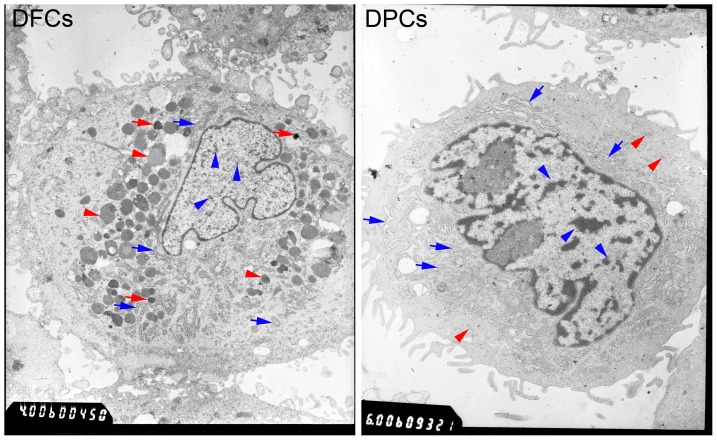
Ultrastructural characterization of DFCs and DPCs. Homogeneous electron-dense granules in DFCs indicated by red arrows are featured for DFCs. RER and mitochondria can be observed in the cytoplasm of DFCs (blue arrows) and DPCs (blue arrows), however, it is more intense in DPCs. A large number of lysosomes (primary lysosomes and secondary lysosomes) were observed in the cytoplasm of DFCs (red arrow heads). Some cell microfilaments were observed in the cytoplasm of DPCs (red arrow heads). Heterochromatin was observed in the nucleus of DFCs and DPCs (blue arrow heads), but less heterochromatin staining was seen in DFCs (blue arrow heads).

Ultrastructural analysis demonstrated that there are more RER and mitochondria in the cytoplasm of DPCs than DFCs. A large number of lysosomes (including primary and secondary lysosomes) were observed in the cytoplasm of DFCs. Some electron-dense granules adjacent to the RER and the lysosome in DFCs were also observed. Cell microfilaments were observed in the cytoplasm of DPCs. Heterochromatin was observed in the nucleus of both DFCs and DPCs, but was fewer in DFCs (Fig. 2).

### Cell proliferation

**Figure 3:**
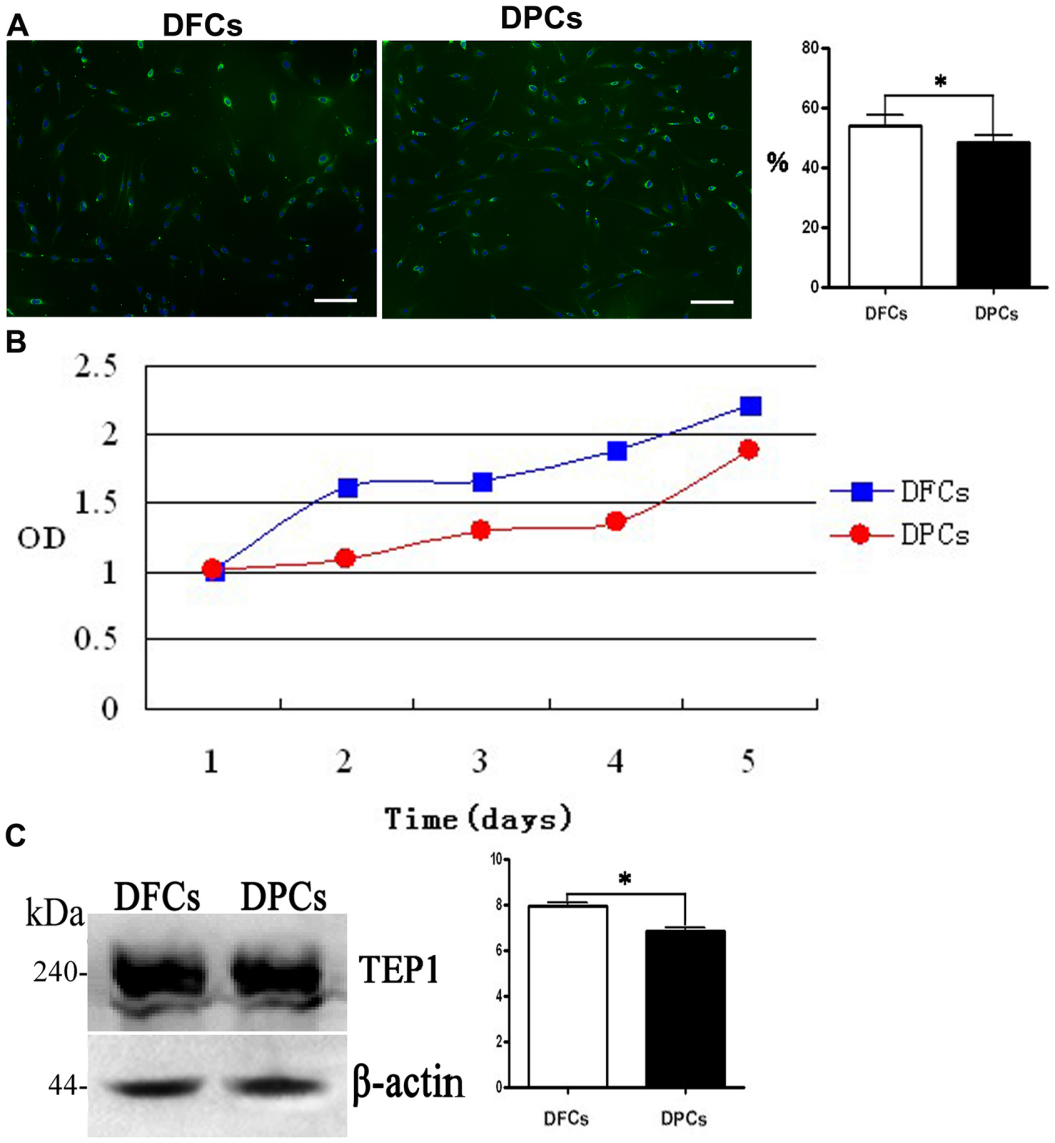
Proliferation potential of DFCs and DPCs. Both cells show high proliferation potential, however, DFCs show a higher potential than DPCs. (A) BrdU labeling, (B) growth curve and (C) TEP1 evaluation via immunofluorescent assay, CCK-8 assay and western blotting. Scale bars  =  100 µm in (A).

BrdU labeling and CCK-8 cell detection protocol showed that both DPCs and DFCs displayed strong proliferation potential, but DFCs exhibited a higher proliferation rate than DPCs (Fig. 3A, B). Both cell phenotypes showed high level of telomerase activity, which was confirmed by western blotting (Fig. 3C). The telomerase activity in DFCs was significantly higher than in DPCs (*p*<0.05) (Fig. 3C).

### Immunophenotypic characterization

**Figure 4:**
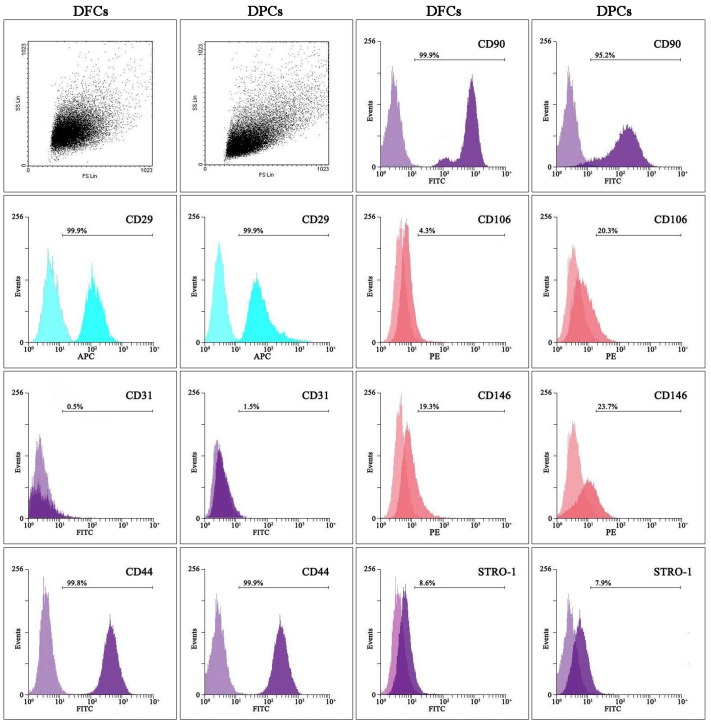
Analysis of cell surface antigens. Flow cytometry analysis indicated that DFCs and DPCs are of mesenchymal origin (positive for CD29, CD44, CD90 and negative for CD31) and DFCs and DPCs are stem cells (positive for CD146 and STRO-1). DPCs are more capable of embryologic vascalogenesis as confirmed by relatively higher expression of CD146 in DFCs. DPCs were positive for the endothelium antigen, CD106 and DFCs were negative for CD106, suggesting that DPCs have a stronger potential to differentiate into vascular endothelial cells.

The immunophenotypic characterization was performed using flow cytometry. Both DFCs and DPCs were positive for STRO-1 (stromal stem cells marker), CD29 (mesenchymal cell marker), CD44 (mesenchymal cell marker), CD90 (mesenchymal cell marker) and CD146 (mesenchymal stem cell marker [15]). The expression of STRO-1 was above 6% in DFCs and DPCs, and statistically significant differences were observed not between the two cell types. The expression of embryologic vasculogenesis marker, CD146 was higher in DPCs than DFCs. The expression of CD29, CD44 and CD90 was above 90% in both cells. Moreover, both cells were negative for the leucocyte precursor marker CD31. However, DPCs were positive for the endothelium antigen, CD106 but DFCs were negative for CD106 staining (Fig. 4).

### Detection of multipotential differentiation potential

**Figure 5:**
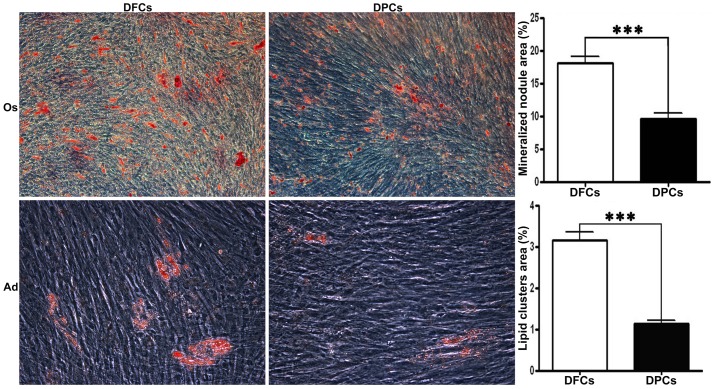
Multipotential differentiation of DFCs and DPCs. After being cultured in osteogenic medium (Os) for 25 days, mineralized nodules were stained with alizarin red. DFCs were more mineralized compared to DPCs (*p*<0.05). Oil red O staining was used to assess the formation of lipid droplets in the adipogenic cultures. DFCs formed more lipid droplets compared to DPCs (*p*<0.05). Scale bars  =  100 µm.

After being cultured in either osteogenic or adipogenic media for 25 days, DFCs formed more mineralized nodules and lipid droplets compared to DPCs (*p*<0.05) (Fig. 5).

### Differential expression of proteins in DFCs and DPCs

**Figure 6:**
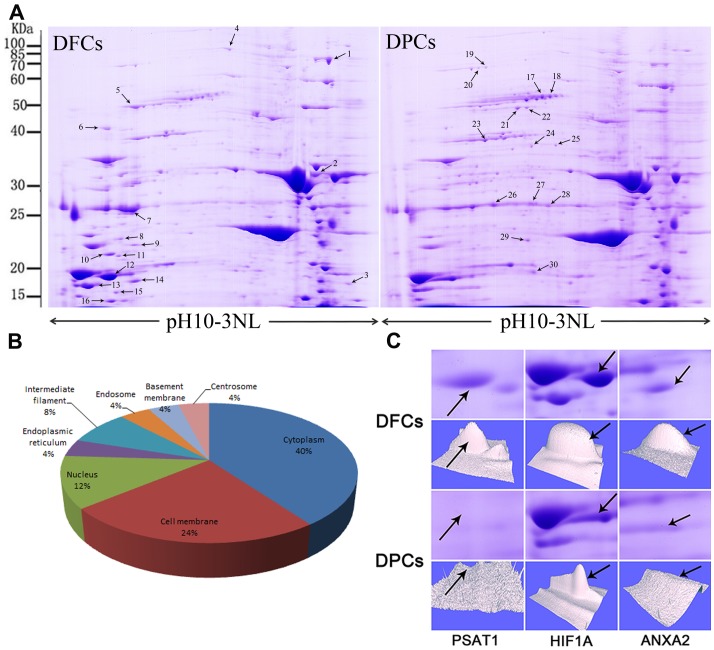
Proteomics analysis of differentially expressed proteins in DFCs and DPCs. (A) Representative 2-DE maps of human DFCs and DPCs. (B) The listed proteins are localized in the cytoplasm (40%), nucleus (12%), intermediate filaments (8%), cell membrane (24%), endoplasmic reticulum (4%), endosome (4%), basement membrane or centrosome (4%). (C) Expression profile of three significantly altered proteins (#10, #12 and #14). The selected area was enlarged, and arrows indicate each protein spot or its theoretical location. 3D view showing the alteration of the expression of three proteins was generated by PD-Quest software.

Protein extracts of DFCs and DPCs were separated by 2-dimensional electrophoresis. Representative 2-D maps for a subsample of six pairs of samples that were matched by the PD-Quest software are shown in Fig. 6A. Differential expression of proteins was considered as statistically significant (*p*<0.05) when the intensity alterations were over 1.5-fold and was also observed in more than two experiments. By applying these criteria, a total of 30 spots were identified as being differentially expressed. Among these, 16 proteins were expressed at higher levels in DFCs while the rest were expressed at higher levels in DPCs (Fig. 6A).

30 spots were selected and analyzed using MALDI-TOF tandem mass spectrometry. The MS/MS data were queried using the search algorithm MASCOT against the ExPASy protein sequence database. Proteins were identified based on these criteria: pI, MW, the number of matched-peptides and MOWSE score. A total of 12 proteins from 30 spots were identified and detailed information about these proteins is listed in Table 2. The identified proteins were classified into different groups based on their sub-cellular localization (Fig. 6B). The most differentially expressed proteins in these two cells include phosphoserine aminotransferase (PSAT1), Isoform 2 of Hypoxia-inducible factor 1-alpha (HIF1A) and Isoform 1 of Annexin A2 (ANXA2) (Fig. 6C).

**Table 2:** Proteins identified by MALDI-TOF-MS.

Spot Number	Protein Name	Accession No.^a^	Protein Score^b^	Protein Score C.I.%
1	HNRNPD 30 kDa protein	IPI00964648	77	99.814
2	VIM Vimentin	IPI00418471	67	98.181
3	LDHA Isoform 1 of L-lactate dehydrogenase A chain	IPI00217966	82	99.938
5	LMNA Isoform A of Prelamin-A/C	IPI00021405	122	100
6	ZNF484 cDNA FLJ56482, highly similar to Zinc finger protein 484	IPI00184544	68	98.487
7	ENO1 Isoform alpha-enolase of Alpha-enolase	IPI00465248	103	100
9	GOT1 Aspartate aminotransferase	IPI00922421	62	94.754
10	PSAT1 Phosphoserine aminotransferase	IPI00001734	154	100
12	ANXA2 Isoform 1 of Annexin A2	IPI00455315	141	100
13	LDHA Isoform 1 of L-lactate dehydrogenase A chain	IPI00217966	134	100
14	HIF1A Isoform 2 of Hypoxia-inducible factor 1-alpha	IPI00332963	65	97.117
21	MSN Moesin	IPI00219365	61	93.242

All protein spots were identified by Applied Biosystems 4800 Proteomics Discovery System.

a) Accession Numbers were derived from the IPI-HUMAN V3.52 database.

b) Protein score > 60 is considered to be confidence.

### Odontogenic differentiation of DFCs and DPCs *in vitro*

#### SEM Observation

**Figure 7:**
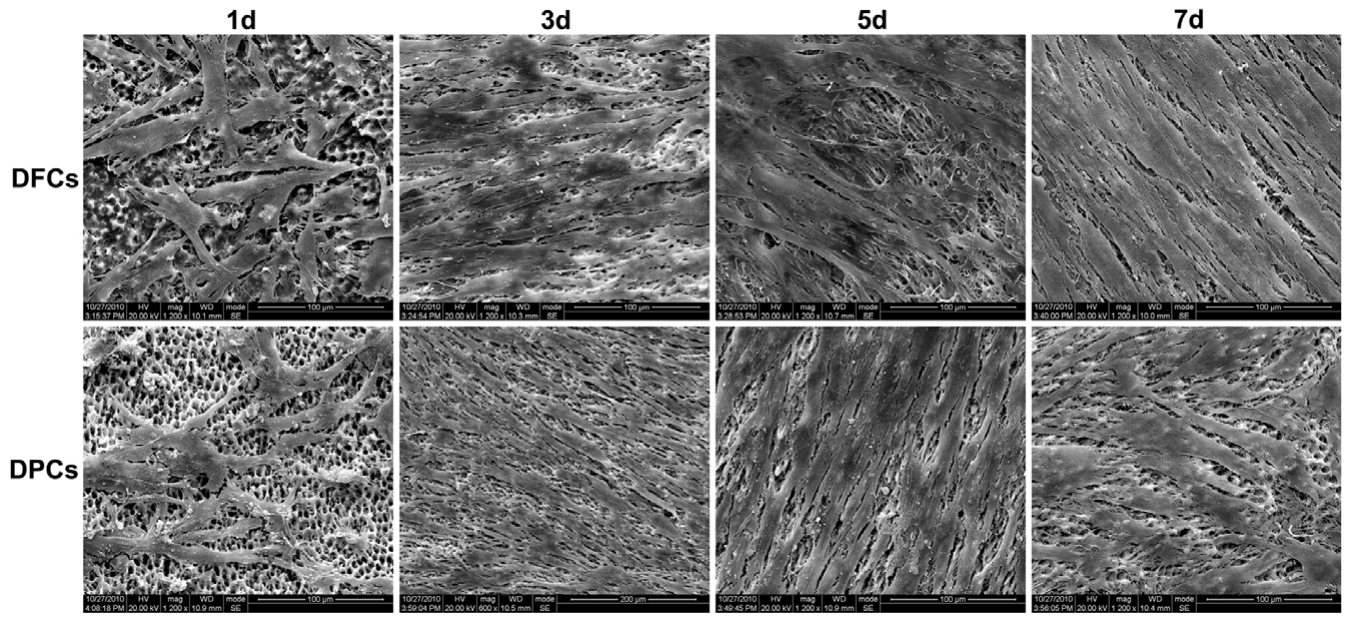
SEM examination of the growth of DFCs and DPCs on human TDM. Cell number was low on day 1; on day 3, dentinal tubules were disappearing due to coverage by cells. On day 5 and 7, no dentinal tubulus were observed and multilayer cells were observed. Scale bars  =  100 µm.

Cell morphology of DFCs and DPCs was examined using SEM after they had been seeded on TDM for 1, 3, 5, and 7 days. On day 1, cell number was relatively low, the attached cells showed a polygonal shape. On day 3, cells proliferated on TDM, resulting in the coverage of dentinal tubules. On day 5 and 7, very few dentinal tubules were observed due to the high density of cells and their extracellular matrix. Furthermore, multiple layers of cells were observed on the surface of TDM. There were no significant differences between the two kinds of cells on day 5, but on day 7, more directional arrangement of DFCs was observed on TDM (Fig. 7).

#### qRT-PCR and western blotting

**Figure 8:**
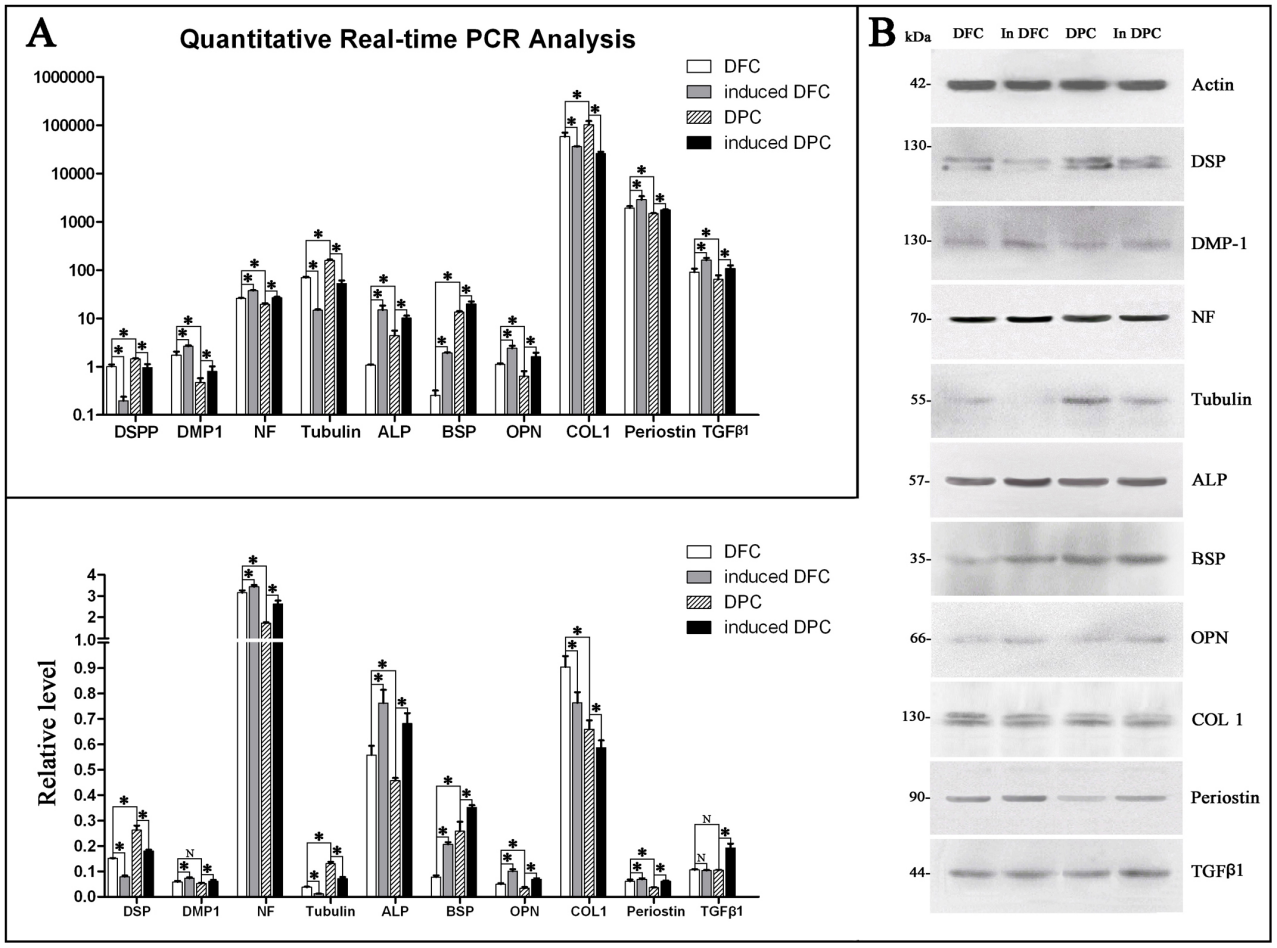
Expression of markers related to odontogenic differentiation in DFCs and DPCs. The odontogenic genes and proteins were detected in DFCs, iDFCs, DPCs and iDPCs by (A) qRT-PCR and (B) western blotting. * *p*<0.05. iDFCs:DFCs induced in TDM, iDPCs:DPCs induced in TDM.

Results from both qRT-PCR and western blotting showed that both cells can express markers related to dentinogenesis, including DSPP/DSP, DMP1, NF, Tubulin, ALP, BSP, OPN, COL1, periostin and TGF-β1 (Fig. 8). Importantly, expression of DMP1, NF, ALP, OPN, COL1 and periostin was higher in DFCs than in DPCs. Similar results were obtained when induced DFCs (iDFCs) and induced DPCs (iDPCs) with TDM were compared. Moreover, higher expression levels of DSPP/DSP, Tubulin and BSP were detected in DPCs compared to DFCs, and higher expression levels of DSPP/DSP, Tubulin, BSP and TGF-β1 were detected in iDPCs compared to iDFCs. The expression of DMP1, NF, ALP, BSP, OPN, periostin and TGF-β1 was up-regulated in iDFCs compared to DFCs, and the expression of the above genes and proteins was also up-regulated in iDPCs compared to DPCs. However, the expression of DSPP/DSP, Tubulin and COL1 was down-regulated in iDFCs compared to DFCs, and the expression of the same genes was also down-regulated in iDPCs compared to DPCs.

### *In vivo* odontogenic differentiation of DPCs and DFCs

**Figure 9:**
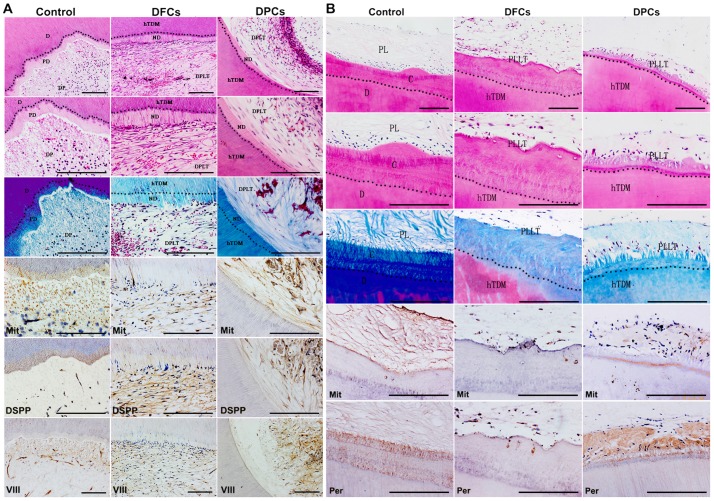
Odontogenic differentiation of DFCs and DPCs *in vivo*. Both DFCs and DPCs contribute to the regeneration of (A) pulp-dentin complex inside TDM and (B) of PDL-cementum complex outside TDM. D: dentin, PD: predentin, DP: dental pulp, ND: neo-dentin, DPLT: dental pulp-like tissue, hTDM: human treated dentin matrix, C: cementum, PL: periodontal ligament, PLLT: periodontal ligament-like tissue. Scale bars  = 100 µm.

After 8 weeks of growth, the grafts were harvested and subjected to histological analysis. Results showed evidence of neo-dentin and formation of dental pulp-like tissues inside the grafts (Fig. 9A). Periodontal ligament-like tissues were observed on the surface of the grafts (Fig. 9B). There was no qualitative difference between the two cell-groups. Immunohistochemistry (IHC) analysis showed that neo-dentin tissues were positive for DSP, a marker of dentin. The expression of Factor VIII was observed in the loose connective tissue inside TDM. The neo-periodontal ligament-like tissues were positive for periostin, a periodontogenesis marker. The cells surrounding the regenerated tissue were partly positive for human mitochondria (Fig. 9 A, B).

## Discussion

As the precursor cells of odontoblasts, DPCs can differentiate into odontoblasts [16], [17]. As the origins of periodontium [18], DFCs can differentiate into various types of periodontium cells including cementoblasts/osteoblasts [19], neurons and adipocytes [10]. However, their differentiation requires induction from the surrounding micro-environment during development.

In this study, DFCs and DPCs were obtained from human dental follicle and dental papilla at the crown-forming stage. Abundance of RER and mitochondria in DFCs and DPCs indicated that cells have a strong ability for protein synthesis and secretion [20]. Less heterochromatin in the nuclei of DFCs compared to that of DPCs indicated that DFCs were more pluripotent than DPCs [21], [22]. Moreover, a higher proliferation rate for DFCs compared to DPCs may be due to higher telomerase activity in DFCs [5]. Telomerase is known to play key roles in cellular apolexis and longevity. Undifferentiated embryonic stem cells (ESCs) generally show strong telomerase activity, but the telomerase activity gradually decreases in differentiating or differentiated ESCs [23]. These results imply that perhaps, DFCs possess stronger undifferentiated characteristics than DPCs [23]. Additionally, an increase in telomerase activity in the adult stem cells influences their ability for self-renewal, sustaining cell division and providing chromosomal stability [24]. Our studies reveal that DFCs are more capable of differentiating along multiple mesenchymal lineages and maintaining their ability for self-renewal [5], [7], [8].

The immunophenotypic characterization showed that both DFCs and DPCs exhibit expression patterns similar to bone marrow-derived stem cell (BMSC) [25]-[27]. Both cells were positive for mesenchymal antigens (STRO-1, CD29, CD44, CD90 and CD146), negative for haematopoietic antigens (CD31). Significantly higher expression of CD146 and CD106 in DPCs compared to DFCs suggested that DPCs were more capable of vascalogenesis [28]. The analysis of stem cell surface epitopes revealed that DFCs and DPCs retained the characteristics of mesenchymal stem cells, and both of them can differentiate along multiple lineages. However, our results indicated that DPCs were better at vascular differentiation. This was consistent with the fate of DPCs as the precursor cells of dental pulp cells, suggesting they are more likely to differentiate into vascular endothelial cells. Furthermore, both DFCs and DPCs can cause mineralization, but we observed a stronger osteogenic differentiation potential for DFCs than DPCs may due to DFCs are the precursors for periodontium cells and DPCs are precursors dental pulp cells [9], [10], [16], [17], [19], [29], [30].

In this study, 12 identified proteins were mainly related to protein secretion and protein synthesis. For instance, HSP90B1 endoplasmin is the key factor in signal transduction, protein folding, protein degradation and morphological evolution. PSAT1 is essential for stimulation of osteoclastogenesis and cell proliferation [31]. ANXA2 is an important osteoporosis susceptibility gene, which is involved in osteoporosis in humans [32]. HIF1A is a master transcriptional regulator of the adaptive response to hypoxia and it activates the transcription of over 40 genes under hypoxic conditions [33]-[35]. One of the higher expressed protein in DPCs is moesin (MSN), it localizes to filopodia and other membranous protrusions, which are important for cell-cell recognition, signaling and cell movement. The up-regulated proteins in DFCs indicated that DFCs may have a greater ability to regulate bone formation and resorption, and to promote cell survival in a hypoxic environment. These proteins prompt DFCs to adapt to a hypoxic environment in the transplanted region, establish blood supply, and subsequently promote cell survival and form new organization.

Recently, accumulating evidence suggests that the specialized microenvironment can regulate cell differentiation [36], [37]. Previous studies have demonstrated that the cocktail of soluble factors released from developing tooth germ can effectively provide suitable odontogenic microenvironment to contribute to tooth development and regeneration [36], [38]. The molecules involved in signaling pathways change continuously in the local microenvironment during tooth development [36], [39]. During tooth development, both DFCs and DPCs have the chance to contact the developing dentin, which is rich in key molecules involved in tooth development. This indicates that perhaps, dentin regulates the differentiation of DFCs and DPCs. TDM is a bioactive material derived from natural dentin tissue, and it contains large amount of dentinogenic factors [6]. Sufficient exposure of dentinal tubules and loosening of fiber bundles at intertubular and peritubular dentins releases proteins and other factors, which results in the improvement of cell differentiation [6], [7]. The differentiation of DPCs to odontoblasts was accelerated inside dentin when the dentin tissue was contacted, while the differentiation of DFCs to periodontal cells was stimulated on the outside surface of dentin. Hence, the dentin matrix is considered as a key microenvironment factor for the differentiation of DPCs and DFCs during tooth development. In addition, hydroxyapatite (HA)/tricalcium phosphate (TCP) has been used as an inducting micro-environment and as a scaffold in dental tissue regeneration [40], [41]. However, our previous studies have suggested that TDM presents better biocompatibility than HA/TCP [6], [7]. More importantly, it is TDM but not HA/TCP that contributes to the formation of complete dentin tissues *in vivo* [5]-[7]. Therefore, compared to soluble factors from tooth germ and HA/TCP, TDM is a better microenvironment for inducing the differentiation of DFCs and DPCs during tooth development or regeneration.

This study found that both DFCs and DPCs express odontogenic, neurogenic and peridontogenic markers. For example, DSPP and DMP1 are the specific markers of odontoblast differentiation [42]. DSPP gene encodes for two major non-collagenous dentin matrix proteins: DSP and dentin phosphoprotein (DPP). Studies have shown that DSPP splits into DSP and DPP in cells. DSP is secreted in the predentin, while DPP is secreted at the mineralization front and retained in the mineralized dentin [43]. Higher expression level of DSPP in DPCs than DFCs suggests higher dentinogenesis ability of DPCs [44], [45]. The results revealed that the expression of DMP1 in DFCs and DPCs was up-regulated in presence of TDM, but the expression of DSPP was down-regulated in DPCs and DFCs. The down-regulation of DSPP occurs because the formation of mineralized dentine during odontoblasts differentiation causes breakdown of DSPP and production of DSP and DPP. The down-regulation of DSP indicated that cells began to differentiate into odontoblasts and were secreting DSP into predentin (extracellular matrix) [44].

Neural differentiation of mesenchymal cells is a prerequisite for tooth development and regeneration. This study found that DFCs express lower levels of Tubulin and higher levels of NF compared to DPCs. This implies that both DFCs and DPCs have the potential to differentiate into neural-like cells, but DPCs were at an earlier stage of neural differentiation than DFCs [46], [47]. Our results showed that expression of NF was up-regulated but Tubulin was down-regulated for both DFCs and DPCs after induction of TDM, which implied that TDM promoted the differentiation of DFCs and DPCs into neurocytes [48], [49]. The distribution of the pulp nerves is crucial for the regeneration of functional tooth with similar neural reactions as the normal tooth. Therefore, DFCs and DPCs may provide a promising approach in neural differentiation of tooth development and regeneration.

Mineralization is an important event during tooth development and regeneration. ALP plays a vital role in the formation of calcified tissue and extracellular matrix metabolism [50]. OPN is a relatively earlier marker of osteogenic differentiation [51]. BSP is one of the late markers of mineralized tissue differentiation [52]. TGFβ1 participates in TGFβ pathway related to odontoblast proliferation and differentiation [53], [54]. DFCs expressed higher ALP and OPN but lower BSP than DPCs, which suggested that the DPCs were at a more mature stage of osteogenesis. Moreover, as our results demonstrate the beneficial effects of TDM in promoting expression of ALP, OPN, BSP and TGFβ1, we conclude that dentin matrix plays an important role in mineralization of DFCs and DPCs during tooth development.

COL1 and periostin are the key markers of periodontium development and formation [13]. The expression levels of COL1 and periostin were detected in both DFCs and DPCs, however, the expression levels were higher in DFCs. This is because DFCs contains precursor cells for periodontal cells [55], [56]. This study also provided evidence that DPCs can differentiate into periodontal cells. It is known that COL1 is involved in the formation of extracellular matrix components, it is actively expressed in the first proliferation period and then gradually down-regulated during subsequent cell differentiation [57]. In this study, concomitant with TDM induction, COL1 was down-regulated in DPCs and DFCs. This implies that these cells maybe undergoing cell differentiation after the first proliferation period [57]. Hence, TDM has a positive influence on the differentiation of DFCs and DPCs.

To evaluate the odontogenic characteristics of these two cell types *in vivo*, DFCs or DPCs/TDM constructs were subcutaneously implanted into nude mice for 8 weeks. With either cell type, formation of neo-dentin, dental pulp-like tissues, periodontal ligament-like tissues and cementum-like tissues was observed. The cells around the regenerated tissues were positive for human mitochondria, indicating that exogenous DFCs or DPCs participated in the regeneration of new tissue. DFCs are thought to contain precursor cells for cementoblasts, periodontal ligament cells and osteoblasts [56], [58]. In this study, we also demonstrated that TDM induces DFCs to differentiate into odontoblasts [5]-[7], [13]. Although DPCs have been credited as the precursors of odontoblast, this study is the first to find that with the induction of TDM, DPCs contribute to the regeneration of cememtum and periodontal ligament-like tissues. The neo-periodontal ligament-like tissues were positive for periostin and Factor VIII, indicating that TDM can induce the differentiation of DPCs into periodontal cells or endothelial cells [13], [59], [60]. Therefore, DFCs and DPCs have odontogenic characteristics and they have similar odontogenic differentiation potential *in vivo*, which is consistent with the results from the analysis of proteins differentially expressed proteins. Hence, it can be concluded that although the organ germ cells are differentiated, they may still retain the information for organ development. Moreover, they can subsequently differentiate into the corresponding cells and form the corresponding tissues. A comparison of the functions of DFCs and DPCs, based on our study and the previously published reports, is summarized in Table S1. A comparison with other non-secretory cells will help us to fully understand the mechanism by which DFCs and DPCs contribute to odontogenesis, and these await further investigation in future studies.

However, it is critical to note that DFCs and DPCs are a heterogeneous population of cells, and are not pure selected cells. Additionally, these expanded cells contain mixed populations of cells in terms of the stage of cell maturity along the differentiation pathway. The difference between DFCs and DPCs is the site of isolation and they are not a pure population of a single cell type. In summary, this study finds that under the effect of TDM, DFCs are capable of differentiation into odontoblasts to form dentin-like tissues, while DPCs can differentiate into periodontal cells to form the cementum-periodentium-like tissue. The *in vitro* and *in vivo* data presented in this study reveals that DFCs can substitute DPCs in the regeneration of dentin-like tissues. However, a more detailed understanding of the mechanisms involved in differentiation of DFCs and DPCs during tooth development and regeneration is required.

## Supporting Information

Table S1 Summarization and comparison of the functions of DFCs and DPCs.
(DOCX)
