# Supplementary material for: Comparison of Odontogenic Differentiation of Human Dental Follicle Cells and Human Dental Papilla Cells
Source: PLoS One. 2013 Apr 19;8(4):e62332. doi: 10.1371/journal.pone.0062332 (PMC3631153; doi:10.1371/journal.pone.0062332)
Supplement: Table S1 — Summarization and comparison of the functions of DFCs and DPCs. (DOCX) [file pone.0062332.s001.docx]

**Table S1 Summarization and comparison of the functions of DFCs and DPCs**

|  | Previous knowledge | | Our findings | |
| --- | --- | --- | --- | --- |
|  | Target cells | Target tissue | Target cells | Target tissue |
| DFCs | periodontal cells | periodontium | periodontal cells | cementum-periodentium-like tissue |
|  |  |  | odontoblasts | dentin |
| DPCs | odontoblasts | dentin | odontoblasts | dentin |
|  | dental pulp cells | dental pulp | periodontal cells | cementum-periodentium-like tissue |
